# Structural and functional adaptation of *Haloferax volcanii* TFEα/β

**DOI:** 10.1093/nar/gkx1302

**Published:** 2018-01-04

**Authors:** Fabian Blombach, Darya Ausiannikava, Angelo Miguel Figueiredo, Zoja Soloviev, Tanya Prentice, Mark Zhang, Nanruoyi Zhou, Konstantinos Thalassinos, Thorsten Allers, Finn Werner

**Affiliations:** 1 Institute for Structural and Molecular Biology, Division of Biosciences, University College London, London, UK; 2 School of Life Sciences, University of Nottingham, Queen's Medical Centre, Nottingham NG7 2UH, UK

## Abstract

The basal transcription factor TFE enhances transcription initiation by catalysing DNA strand-separation, a process that varies with temperature and ionic strength. Canonical TFE forms a heterodimeric complex whose integrity and function critically relies on a cubane iron-sulphur cluster residing in the TFEβ subunit. Halophilic archaea such as *Haloferax volcanii* have highly divergent putative TFEβ homologues with unknown properties. Here, we demonstrate that *Haloferax* TFEβ lacks the prototypical iron-sulphur cluster yet still forms a stable complex with TFEα. A second metal cluster contained in the zinc ribbon domain in TFEα is highly degenerate but retains low binding affinity for zinc, which contributes to protein folding and stability. The deletion of the *tfeB* gene in *H. volcanii* results in the aberrant expression of approximately one third of all genes, consistent with its function as a basal transcription initiation factor. Interestingly, *tfeB* deletion particularly affects foreign genes including a prophage region. Our results reveal the loss of metal centres in Hvo transcription factors, and confirm the dual function of TFE as basal factor and regulator of transcription.

## INTRODUCTION

The RNA polymerase (RNAP), auxiliary factors and molecular mechanisms of transcription are conserved between archaea and eukaryotes. Many Archaea inhabit extreme environments and their enzymes have adapted to cope with conditions of high temperature or salinity, while the underlying changes of protein chemistry enabling this tolerance remain poorly understood. The molecular engineering challenges of initiating transcription in a start site-specific fashion are independent of the type of RNAP and the domain of life. RNAPs need to engage with the promoter region of the double-stranded DNA template, the two DNA strands need to be separated, the template strand loaded into the RNAP active site, and individual NTP substrates bound in a geometry conducive to catalysis. All cellular RNAPs depend on additional basal transcription factors to facilitate this process. In archaea three basal transcription factors (TBP, TFB and TFE) assist RNAP during initiation. TBP and TFB bind to the TATA and BRE motifs and are necessary and sufficient for RNA polymerase recruitment to the promoter and transcription initiation. TFE is not strictly required for transcription *in vitro*, but stimulates transcription initiation by enhancing open complex formation (OC), i.e. DNA melting and template loading (1).

**Figure 1.**
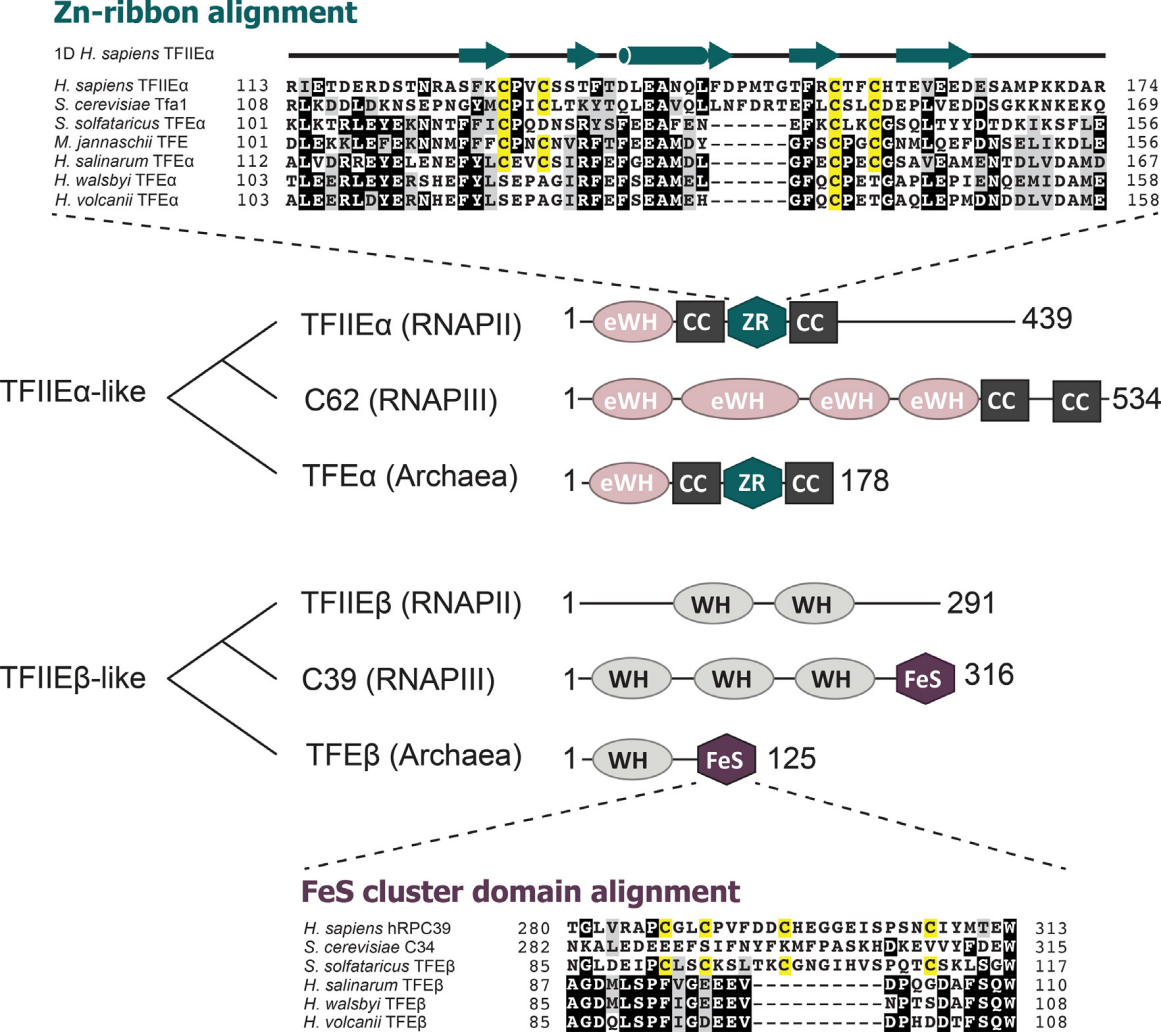
Overview over the domain organization of archaeal TFE subunits and related eukaryotic proteins. TFEα-related proteins are composed of extended winged-helix domains (eWH), a zinc ribbon domain (ZR) and coiled coil helices (CC) (3–5,55). TFEβ-related proteins are composed of winged-helix (WH) and iron-sulphur cluster domains (FeS). Sequence alignments of the two metal binding domains (ZR and FeS) reveal the loss of cysteine residues (highlighted in yellow) required for metal coordination in *H. volcanii* TFEα and TFEβ. Key to sequences: *H. sapiens* TFIIEα (NCBI accession: P29083), *S. cerevisiae* Tfa1 (EDN59878), *S. solfataricus* TFEα (AAK40605), *M. jannaschii* TFE (Q58187), *H. salinarum* TFEα (AAG19231), *H. walsbyi* TFEα (ERG94676), *H. volcanii* TFEα (ELK55987), *H. sapiens* C39 (EAX10240), *S. cerevisiae* C34 (P32910), *S. solfataricus* TFEβ (AAK41220), *H. salinarum* TFEβ (WP_010902769), *H. walsbyi* TFEβ (ERG96516), *H. volcanii* TFEβ (ADE03923). The secondary structure of the *H. sapiens* TFIIEα ZR domain is based on Okuda *et al.* (43).

Both monomeric and heterodimeric TFE variants have been characterized in archaea. In the crenarchaeon *Sulfolobus solfataricus* (Sso) TFE is a heterodimer consisting of TFEα and TFEβ subunits (2). TFEα encompasses an N-terminal extended winged helix-turn-helix (eWH) domain (3) and a C-terminal Zn-ribbon domain (ZR) that is flanked by coiled-coil helices (4,5) (Figure 1). TFEβ encompasses an N-terminal canonical winged helix-turned-helix (WH) and a C-terminal domain coordinating a structural cubane [4Fe-4S] cluster (2). The cluster is highly sensitive to oxidation and its integrity is critical for the stability of TFEα/β and its function (2). The broad phylogenetic distribution of genes encoding TFEα and TFEβ homologues in archaeal genomes suggests that these genes were present in the last common ancestor of all archaea (1). While TFEα is conserved in the vast majority of archaeal species, several archaeal lineages appear to have lost the TFEβ gene (*tfeB*). Canonical *tfeB* genes are conserved in all cren- and thaumarchaeal genomes (6). More divergent putative *tfeB* homologs have also been identified in different euryarchaeal lineages (Methanomicrobiales, Archaeoglobales and Halobacteriales), while the gene is absent altogether from other euryarchaea like *Methanocaldococcus jannaschii* and *Pyrococcus furiosus* (6). Biochemical studies have shown that in these organisms, a monomeric archaeal TFEα variant facilitates transcription (7,8).

Archaeal TFE has two different heterodimeric paralogues in the eukaryotic transcription systems: the basal transcription factor TFIIE and sub-complex hRPC62/39 of human RNAPIII (C82/34 in yeast) (Figure 1). In *S. cerevisiae* both TFIIE subunits are essential for cell viability (9), as are the paralogous RNAPIII subunits C82 and 34 (10,11). Importantly, both eukaryotic complexes appear to have diverged from a primordial TFEα/β-like ancestor by losing either the FeS-cluster coordinating residues (TFIIE subunit β) or the ZR domain (hRPC62) (2,12). In addition to these structural rearrangements occurring at the basis of eukaryotic evolution, in *S. cerevisiae* the C82/34 sub-complex also lost its second metal centre, the Fe-S cluster (2). In absence of high-resolution structures of archaeal TFEβ or eukaryotic hRPC39, the underlying structural adaptation to Fe-S cluster loss in *S. cerevisiae* C34 remains to be understood, but notably the corresponding region in C34 is largely unstructured in the structure of *S. cerevisiae* RNAPIII (13). Thus, in both archaea and eukaryotes, TFE-like factors have undergone major structural adaptations (1).

Functional studies have provided detailed insights into the structural and mechanistic basis of archaeal TFE function during OC formation (1,2,7,8,14). Both TFEα WH and ZR domains are required for the stabilization of the OC (2) via at least two mechanisms, firstly via direct interactions between the WH domain and the nontemplate strand (14) and secondly by an allosteric mechanism that involves the opening of the flexible RNAP clamp (15). The two TFEα domains interact with distinct contact points on the RNAP surface, which likely enables the factor to modulate the position of the flexible clamp; while the N-terminal eWH domain interacts with the coiled-coil of the clamp, the ZR domain contacts the RNAP stalk module, the second major conformationally flexible RNAP domain (15). The function of TFEβ is less well understood, but overall it stabilizes the interaction of TFEα with RNAP and enables all its activities (2). Effectively, the function of TFEα has become dependent on the β-subunit. The molecular mechanism by which OC formation is stimulated is conserved between archaeal TFE and eukaryotic TFIIE (1,5,14,16). The energy requirements for DNA strand separation during OC formation depend in large part on the physicochemical conditions of the environment. Repulsive forces within the phosphate backbones of the DNA strands destabilize the dsDNA helix, but can be counteracted by cations such as Mg^2+^ (17). The effect of human TFIIE on OC formation varies accordingly with KCl and Mg^2+^ concentration (18). Similarly, the effect of archaeal TFEα/β on OC formation increases at higher Mg^2+^ concentrations (F.B. and F.W., unpublished observations).

Haloarchaea thrive in a high salinity environment that is matched by high intracellular K^+^ concentrations, which is thought to have caused global changes in the haloarchaeal proteomes such as low isoelectric points (19). Many studies have shed light on the function of TFB and TBP in the haloarchaeal transcription machinery, which is characterized by its broad expansion of TFB and TBP paralogues that contribute to global regulation of transcription (20–22). In contrast, the function of haloarchaeal TFE has not been investigated thus far. Interestingly, the putative haloarchaeal TFEβ homologs are unusual in that they lack the cysteine residues that chelate the iron-sulphur cluster (Figure 1) (6). This raises the question whether the haloarchaeal homologues function as proper TFEβ subunits in a dimeric complex or whether haloarchaea utilize instead monomeric TFEα variants as described for other members of the euryarchaea. Does the divergent primary sequence of haloarchaeal TFEβ homologues reflect adaptations to the structure and function of haloarchaeal TFE?

Here, we show that the *tfeB* gene from *Haloferax volcanii* (Hvo) does encode a bona fide TFEβ subunit, which does not encompass an FeS cluster and yet still forms a stable complex with TFEα. In addition, the second metal centre of TFE, the TFEα zinc ribbon that is crucial for RNAP binding, is highly degenerate with only a single cysteine residue contributing to zinc binding. This results in a very low affinity for zinc, yet collision induced unfolding experiments demonstrate that zinc binding stabilizes the protein structure. In contrast to *Sulfolobus*, the *Haloferax tfeB* gene is not essential for cell viability. The *tfeB* deletion causes a slow growth phenotype and leads to the transcriptional misregulation of one third of all genes. This substantiates the role of TFE as global regulator in the archaeal domain.

## MATERIALS AND METHODS

### Heterologous expression and protein purification of Hvo TFEα/β

The gene HVO_1174 coding for Hvo TFEα was PCR-amplified and cloned into a modified version of vector pRSF-1b (2) via NcoI and XhoI restriction sites (for all primer and plasmid details see Supplemental Tables S1 and S2, respectively) for the expression of native protein or as a C-terminal His_6_-tag fusion, respectively. HVO_1090 coding for Hvo TFEβ and its variant TFEβ Δ1–72 were cloned likewise via NdeI and XhoI restriction sites into vector pET-21a(+) (Merck). Plasmids were transformed into *Escherichia coli* Rosetta2 (DE3) (Merck) and heterologous expression was carried out in enriched growth medium for 2 h after induction at 37°C. Cells were resuspended in N(500) buffer (25 mM Tris/HCl pH 8.0, 500 mM NaCl, 10 mM MgCl_2_, 100 μM ZnSO_4_, 5 mM 2-mercaptoethanol), disrupted by sonication, cleared by centrifugation and Hvo TFE was isolated by Ni-affinity chromatography.

A TEV-protease cleavage site was introduced between Hvo TFEα and the C-terminal His-tag by the Phusion site directed mutagenesis method (ThermoFisher Scientific). The resulting protein TFEα_TEV_ was expressed and purified as described above. Ni-affinity elution fractions were dialyzed against 20 mM Tris/HCl pH 8.0, 300 mM NaCl, 5 mM 2-mercaptoethanol in the presence of His-tagged TEV protease overnight. TEV protease was removed by Ni-affinity chromatography and successful cleavage was verified by SDS-PAGE.

Size exclusion chromatography was carried out in N(500) buffer with a flow rate of 0.5 ml/min on a Superose 12 hr 10/300 column (GE Lifesciences) with 0.5 ml fractions collected.

### Gene deletion and growth conditions

Growth and transformation of *H. volcanii*, isolation of genomic DNA and construction of deletion mutants was carried out as described previously (23). The *tfeA* gene HVO_1174 is in an operon with HVO_1175 and HVO_1176, which was PCR-amplified (see Supplemental Table S1) and cloned in pTA1744, a derivative of pTA131 (23), to generate pTA1769. A deletion construct pTA1814 was generated using outward facing primers to amplify upstream and downstream flanking regions of *tfeA*, respectively. A novel *Nco*I site was used for insertion of a promoter-less *trpA* selectable marker from pTA1844, generating pTA1858; care was taken to not affect transcription of the remaining operon. pTA1858 was used to transform Hvo strain H53 (*ΔpyrE2 ΔtrpA*, see Supplemental Table S3) and *ΔtfeA::trpA+* colonies were screened as described previously (23). All 129 colonies examined by hybridization were merodiploid and had retained the wild-type *tfeA*. Therefore, *tfeA* is most likely essential.

The genomic region containing the *tfeB* gene HVO_1090 was PCR-amplified and cloned in pTA131 (23) to generate pTA1706. A deletion construct pTA1708 was generated using outward facing primers to amplify upstream and downstream flanking regions of *tfeB*. A novel BglII site was used for insertion of the *trpA* selectable marker from pTA298 generating pTA1709, which was used to transform Hvo strain H53 and *ΔtfeB::trpA+* colonies were screened as described previously (23). 120 of the 160 colonies examined by hybridization were *ΔtfeB*, which was verified for 8 candidates by Southern blot (Figure 5A).

To measure growth of the *ΔtfeB* mutant strains H2644 and H2645 in Hv-YPC broth (23), 250 μl cultures were incubated in 48-well plates at 45°C, with continuous double-orbital shaking at 425 rpm, using a BioTek Epoch2 microplate spectrophotometer. Optical density at 600 nm was measured every 15 minutes and generation times were calculated by curve-fitting to the log phase of the growth curve, as indicated by the straight-line portion between perpendicular marks in Figure 5B. The standard errors of generations times across replicates = 0.07 h for wild-type and 0.13 h for *ΔtfeB* mutant. To complement the *ΔtfeB* mutation *in trans*, a KpnI–NotI fragment of pTA1706 containing the *tfeB* gene and its promoter was cloned in the shuttle vector pTA354 (24) to generate pTA2050, which was used to transform the *ΔtfeB* mutant H2644 and its parent H53; the pTA354 empty vector was used as a control. Growth was measured in a microplate spectrophotometer as described above (Figure 5C). Raw growth data are provided in Supplemental Table S4.

### RNA-seq RNA isolation

Total RNA was isolated from *H. volcanii* using the TRIzol method. 50 ml of cells grown in Hv-YPC broth to early log phase (OD_650_ = 0.2) were resuspended in 250 μl of unbuffered spheroplast solution (1 M NaCl, 27 mM KCl, 15% sucrose, pH 7.5) (23). 750 μl of TRIzol LS (Invitrogen) was added and mixed by pipetting. 200 μl of chloroform (Sigma) was added and vortexed for 15 s, then centrifuged at 20 000 x *g* for 15 min. The upper phase was transferred to a fresh tube, 500 μl of isopropanol was added and incubated for 10 min at room temperature. RNA was pelleted by centrifugation at 20 000 x *g* for 10 min, washed with 75% ethanol, air-dried and resuspended in 50 μl of RNase-free dH_2_O. Total RNA quality was assessed using the RNA 6000 Nano Kit (Agilent) on an Agilent 2100 Bioanalyzer. Total RNA concentration was measured using Qubit RNA BR assay kit (Life technologies, Q10210). 1 μg of total RNA was used for rRNA depletion using Ribo-Zero rRNA Removal Kit (Illumina). Libraries were prepared using the dUTP method (25) with NEBNext Ultra Directional RNA Library Prep kit for Illumina (New England Biolabs) and library quality was analyzed on a Bioanalyzer High-Sensitivity DNA-chip (Agilent Biotechnologies). Sequencing was performed on the Illumina NextSeq 500 sequencing platform to generate 2 × 75 bp paired end reads using V2 chemistry.

### RNA-seq data analysis

Sequencing reads were trimmed to remove the adaptor parts using Trimmomatic (26) and the quality of the sequencing data was assessed using FastQC (http://www.bioinformatics.babraham.ac.uk/projects/fastqc). Reads were mapped to the Hvo genome using TopHat (27) using parameters –no-novel-juncs –no-mixed –library-type fr-first strand with filtering out reads mapping to tRNA and rRNA genes as well as reads that do not map uniquely. Principal component analysis (PCA) was used to assess biological replicate concordance. RPKM values (reads per kb per million) were calculated using HTseq-count. Data were analyzed using DESeq2 to identify significant differentially expressed genes (*P*_adj_ < 0.01) (28).

In order to calculate transcript abundance, we combined an available transcriptome map of Hvo (29) with recently published transcription start site (TSS) mapping data (30) to create an updated map of Hvo mRNA transcriptome. For 1331 transcription units (TUs) the 5′-end coordinates were adjusted based on mapped primary TSSs. For these cases, the median adjustment was by 5 bp, whereas in 117 cases an adjustment of >100 nt was required, in the vast majority leading to an extension of the TU. Six annotated polycistronic TUs harboured additional internal primary TSSs. In these cases the TU annotation was revised and shortened to the primary TSS position. 62 protein encoding genes with assigned TSSs were not included in the existing TU map (29). This updated map (Supplemental Table S7) was used to estimate expression levels using RSEM in paired end mode and –strandedness reverse for libraries prepared with the dUTP method (31).

The same transcription start site mapping data were used for promoter analysis. DNA sequences were extracted using BedTools getfasta (32) for positions –50 to +10 relative to the TSS and analyzed using the MEME software version 4.11.4 for motif identification (0 or 1 occurrence per sequence, 4–16 bp width, searching given strand only) (33). A 0-order background model based on the composition of the combined *H. volcanii* DS2 genome was employed. Aligned promoter sequences were depicted using WebLogo3 with correction for the genome composition (34). Synonymous codon usage data for Hvo genes have been described previously (19). AT% of genes was calculated using BedTools nuc (32). Data were plotted using R (http:/www.R-project.org) with the LSD package v3.0.

### Native mass spectrometry

For nESI mass spectrometry (MS) proteins were buffer changed to 0.5 M NH_4_ acetate by ultrafiltration. Zn(II)-acetate was added to the samples at 2:1 molar ratio followed by 1 hr incubation at room temperature. All samples were analyzed on a first generation Synapt mass spectrometer (Waters corp., Manchester, UK) (35). Samples were introduced into the mass spectrometer by means of nESI at a concentration of 10–20 μM. For protein unfolding experiments ZipTips (C4, Millipore) were used to buffer exchange samples into 50–70% acetonitrile containing 0.1% TFA according to the manufacturer's protocol. All samples were introduced into the mass spectrometer using *in-house* prepared gold-coated capillaries (borosilicate glass capillaries with dimensions 1.0 mm × 0.78 mm from Harvard apparatus) created using a needle-puller (P-97, Sutter Instrument) and coated with gold using a sputter-coater (SC7620, Emitech), as described previously (36). The mass spectrometer was externally calibrated using a 30 mg/ml solution of caesium iodide. Instrument setting parameters were as follows: capillary voltage 1.2 kV, cone voltage 40 V, source temperature 40°C, extraction cone voltage 1 V, trap collision energy (CE) 6 V, transfer CE 4 V and bias voltage 4 V. For complexes with a mass >25 000 Da, the backing pressure was increased to 4 mbar. For collision induced unfolding (CIU) experiments bias voltage was increased to 17 V, and a CE ramp (6–36 V) in the trap collision cell was used. Mass spectra were analyzed in MassLynx software v4.1 (Waters) and CIU maps were analyzed using Amphitrite (37).

### NMR spectroscopy

Isotopically labeled samples for NMR studies were prepared by growing BL21 (DE3) cells for heterologous expression of protein TFEα_TEV_ in minimal (M9) medium using as nitrogen source ^15^NH_4_Cl (CK Isotopes). The protein was purified as described above by Ni-affinity chromatography, TEV protease cleavage and size exclusion chromatography, but the last step was carried out in an NMR compatible buffer (10 mM HEPES/KOH pH 7.5, 100 mM NaCl, 1 mM DTT).

NMR experiments were performed on a Bruker Avance III 600 MHz spectrometer, equipped with a 5 mm TXI room temperature probe at 25°C. The protein concentration for samples used for NMR studies was typically kept at 0.3 mM in the same buffer as above supplemented with 7% (v/v) ^2^H_2_O. Zn^2+^ was added using a ZnSO_4_ stock solution diluted to a final concentration of 165 and 190 mM corresponding to ∼1:1 molar ratio for TFEα_TEV_ and TFEα_TEV_ C138S, respectively. 2D ^15^N–^1^H HSQC spectra were acquired for each Zn^2+^ concentration. Data were processed using the NMRPipe/NMRDraw (38) package and analyzed with CCPNMR (39).

## RESULTS

### *H. volcanii* TFEα/β forms a stable heterodimer

**Figure 2.**
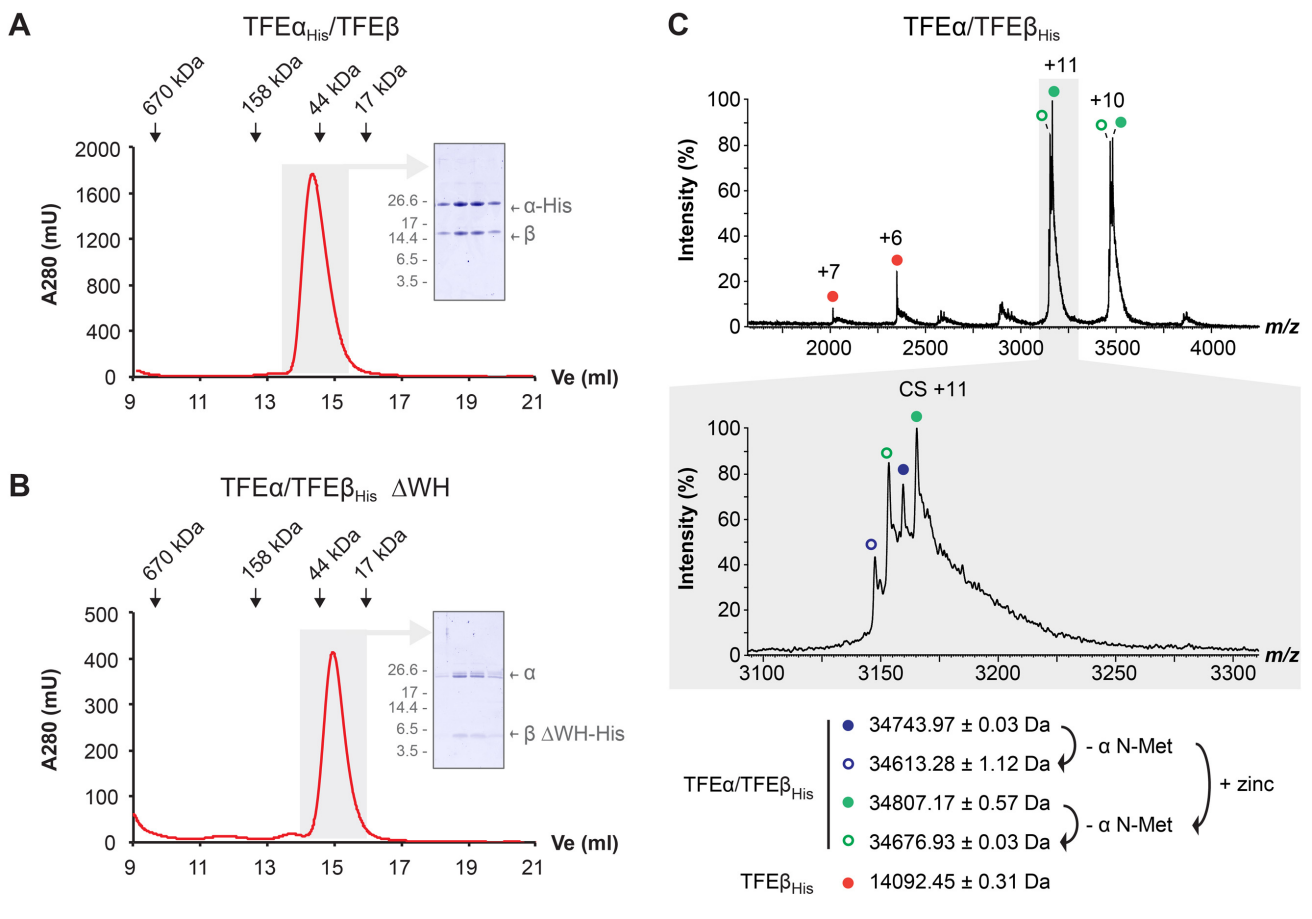
*Hvo* TFEα and TFEβ form a dimeric complex in the absence of an FeS cluster. (**A** and **B**) Size exclusion chromatography of TFEα_His_/TFEβ (A) and TFEα/TFEβ ΔWH_His_ (B) on a superose 12 h 10/30 column. Elution peaks of standard proteins are indicated. Peak fractions (highlighted in gray) were subjected to SDS-PAGE and Coomassie-staining (insert) to show symmetric elution of both subunits. (**C**) nESI mass spectrum of TFEα/TFEβ_His_. The different charge state series observed are labelled with red, blue and green filled circles. Charge state series derived from additional cleavage of TFEα N-Met are labelled with open circles. Highlighted in gray is a zoom over the region that includes charge state (CS) +11. All detected masses indicate the absence of a cubane 4Fe–4S cluster (352 Da).

The euryarchaeon *H. volcanii* (Hvo) harbours ORFs annotated in the arCOG database as TFEα and β subunit-encoding genes (arCOG04270 and arCOG04153, respectively) (40). In order to determine whether their gene products interact, we cloned the two genes *tfeA* (HVO_1174) and *tfeB* (HVO_1090) into compatible plasmids for coexpression in *E. coli*. Either of the two proteins was fused to a C-terminal His-tag in order to facilitate purification using Ni-affinity chromatography. SDS PAGE analysis of the elution fractions revealed that both TFEα and β subunits co-eluted irrespective of which subunit was tagged, suggesting that they form a stable complex. The fractions containing the co-eluting proteins were furthermore subjected to gel filtration (Figure 2). Both TFE α- and β-subunits eluted symmetrically with their predicted mobility, demonstrating that they form a stable heterodimeric TFEα/β complex (Figure 2A). The Sso TFEβ FeS cluster domain is essential for dimer formation, while the WH domain is dispensable. We constructed the corresponding WH deletion variant of Hvo TFEβ and verified its complex formation with TFEα, which confirms that the WH domain is dispensable for dimer formation in Hvo and demonstrates that the domain requirements for dimerization are conserved between Sso and Hvo TFEα/β (Figure 2B).

### Unusual metal centre binding properties of Hvo TFEα/β

FeS clusters are commonly coordinated by cysteine residues, but alternatively arginine, histidine or aspartate residues can substitute for single cysteine residues (41,42). In the case of haloarchaeal TFEβ candidates, the absence of all four cysteine residues suggests that they do not contain FeS clusters (Figure 1). The TFEα ZR domain contributes the second metal centre of archaeal TFEα/β complexes. In the human TFIIEa ZR domain, four cysteine residues chelate a zinc ion (43). Likewise, the majority of archaeal TFEα homologues use four cysteine residues for zinc binding. Histidine, glutamate and aspartate residues have been shown to participate in zinc coordination in place of cysteine, and in some archaeal TFEα variants including Sso one of the four cysteines in the ZR domain has been substituted by an aspartate residue (44). Surprisingly, Hvo TFEα features only a single cysteine residue while the first, second and fourth cysteine positions are substituted by serine, alanine and threonine residues, respectively (Figure 1). This degenerate Hvo TFEα ZR domain has likely lost its ability to coordinate zinc altogether, or involves glutamate or other residues present in the loop of the two β-hairpins that form the canonical ZR motif. Notably the conservation of TFEα cysteine residues differs between the two major groups within the haloarchaea. While some halophilic archaea such as *Halobacterium salinarum* have TFEα variants with all four cysteines conserved, others such as *Haloquadratum walsbyi* TFEα have the same substitutions as in *H. volcanii* with a single conserved cysteine.

In order to characterize the nature of the metal binding properties of *Hvo* TFEα/TFEβ, we carried out native nano electrospray-ionization (nESI) mass spectrometry. The native mass spectrum of TFEα/TFEβ-His confirms the formation of a dimeric complex (Figure 2C). The TFEα/TFEβ-His complex was detected as a set of four peaks of calculated masses of 34 613.28 (± 1.12) Da, 34 743.97 (± 0.03) Da, 34 676.93 (± 0.03) Da and 34 807.17 (± 0.57 Da) due to two different additional masses splitting the signal independently. One split is due to the partial N-Met cleavage of TFEα by methionine aminopeptidase during heterologous expression in *E. coli* (Supplementary Figure S1); this cleavage accounts for a mass difference of 131 Da. The second split is compatible with the presence or absence of a zinc ion, which accounts for an additional mass of 65.38 Da. The spectra revealed a small amount of free TFEβ-His that resulted in a second charge state series with a mass of 14 092.45 (± 0.31) Da coinciding well with the theoretical mass of TFEβ-His (14 090.5 Da, Figure 2C). This suggests that the zinc present in the TFEα/TFEβ complex must be bound by the α subunit – in spite of the degeneracy of the zinc binding motif. We also noted that extended washing lead to complete loss of the heavier species indicative of zinc loss (data not shown).

### Zinc binding stabilizes the degenerate TFEα ZR domain

**Figure 3.**
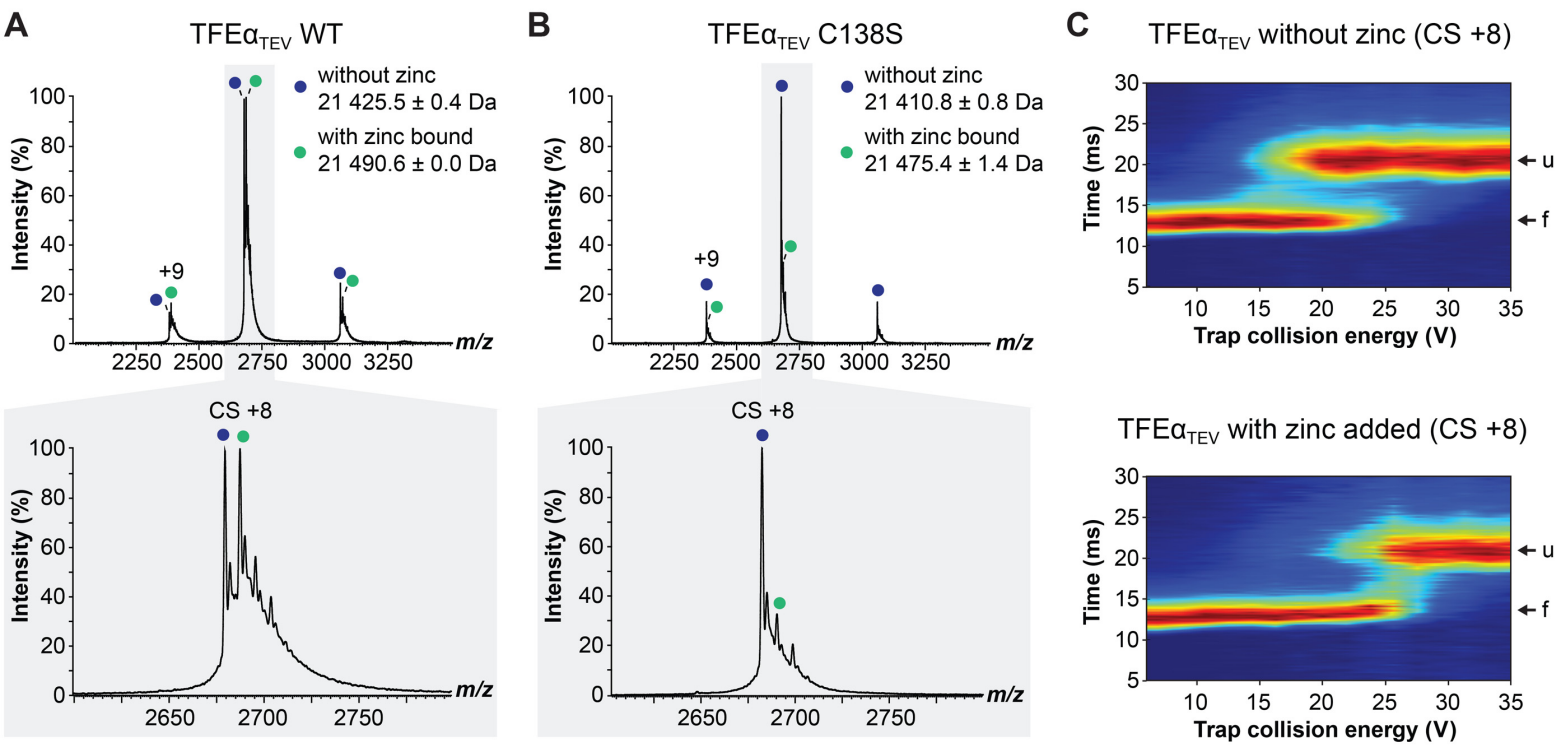
The degenerate zinc-ribbon domain of TFEα is capable of zinc binding. (**A** and **B**) nESI native mass spectrum of monomeric wild type TFEα_TEV_ (A) and TFEα_TEV_ C138S (B) after incubation with zinc. Charge state series of TFEα_TEV_ with and without zinc are labelled with green and blue filled circles, respectively. Highlighted in gray is a zoom over the region that includes charge state (CS) +8. (**C**) Collision induced unfolding experiment of TFEα_TEV_ with and without zinc addition. CS+8 was isolated using the quadrupole and activated in the trap collision cell using a CE ramp (6–36 V) prior to ion mobility separation. The ion mobility cell separated the protein into two populations corresponding to folded (f) and unfolded (u) states.

In order to characterize the binding of zinc to the TFEα ZR domain, we produced a recombinant monomeric TFEα variant (TFEα_TEV_) that allowed the removal of the affinity tag following purification. To prevent zinc loss due to the additional purification steps, we re-incubated the protein with 1 mM ZnSO_4_ before the buffer exchange into NH_4_ acetate compatible with nESI MS. The native mass spectrum of TFEα_TEV_ showed two peaks of similar intensity with a calculated mass of 21 425.5 (± 0.4) Da and 21 490.6 (± 0.0) Da corresponding very well with the theoretical mass of TFEα_TEV_ (21 425.65 Da) and an additional zinc ion (65.38 Da, Figure 3A). In order to characterize any putative zinc chelating residues, we mutated the single conserved cysteine residue in the TFEα ZR domain to serine and recorded its native mass spectrum. The C138S variant showed one major peak with a calculated mass of 21 410.8 ± 0.8 Da corresponding to the theoretical mass of the ligand-free protein (21 409.59 Da) and a very small population with a mass of 21 475.4 (±1.4 Da) matching the theoretical mass of the zinc-bound form (21 474.97 Da, Figure 3B).

**Figure 4.**
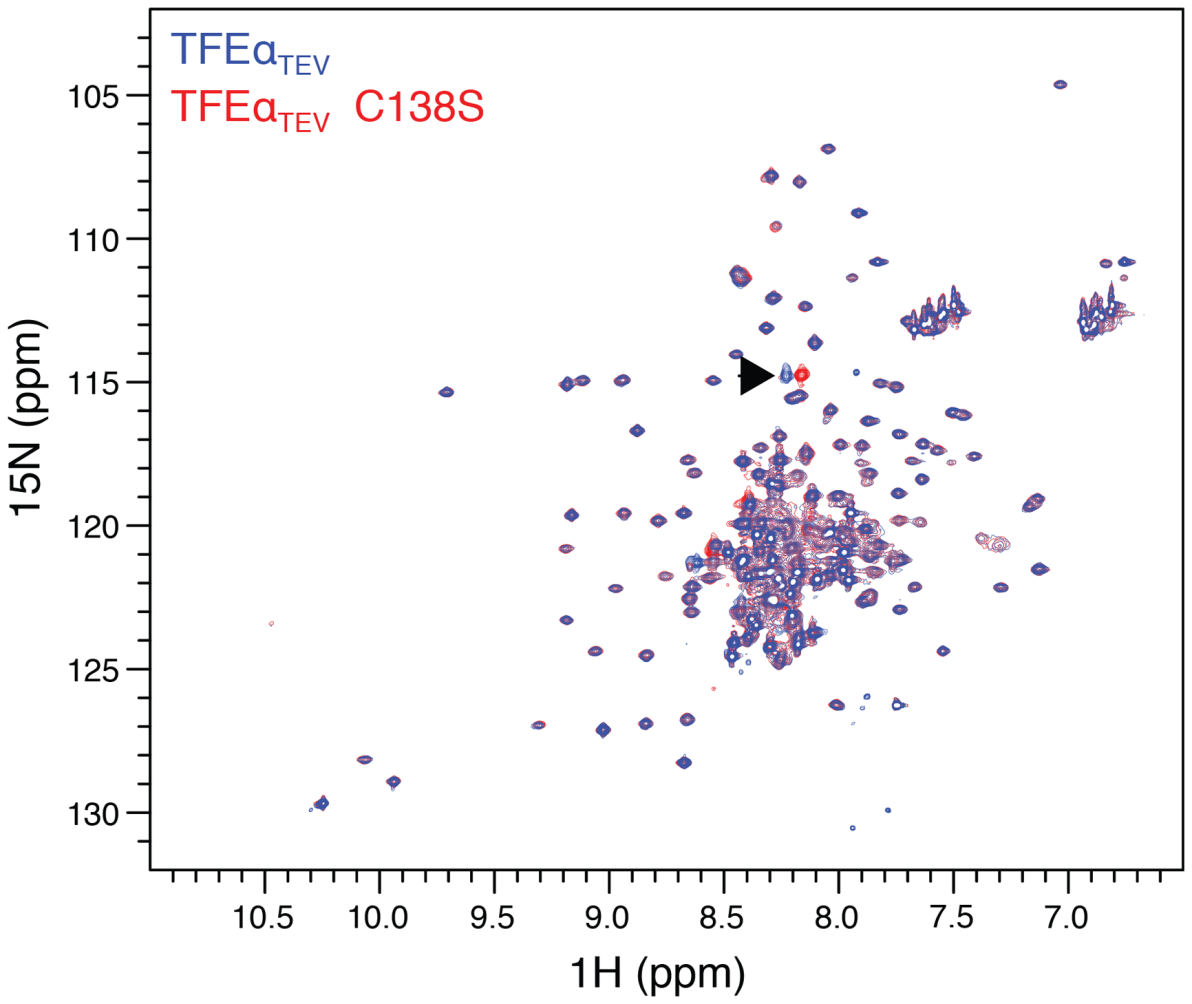
The C138S mutation does not cause larger structural perturbation of TFEα_TEV._ Overlay 2D ^15^N–^1^H HSQC spectra of TFEα_TEV_ (blue) and C138S variant (red) without Zn^2+^. The black arrow indicates the chemical shift perturbation caused by the C138S mutation. All data were recorded at 600 MHz, under the same conditions, number of scans and increments, at pH 7.5, 7% D_2_O, 25°C.

In order to assess whether zinc binding increased the stability of TFEα_TEV_, we carried out collision-induced unfolding (CIU) experiments. CIU combines activation of proteins by voltage to induce protein unfolding coupled with ion mobility mass spectrometry to detect the folding status (45). Ligand-free TFEα_TEV_ appeared in two populations, a faster travelling population (∼15 ms) corresponding to the native folded protein, and a slower travelling population (∼25 ms) equivalent to a more unfolded state triggered by a higher voltage (∼20 V, Figure 3C). To analyze the effect of zinc, we preincubated TFEα_TEV_ with 1 mM zinc acetate, isolated the corresponding peak (21 490.6 Da charge state +8) using the quadrupole mass analyzer, and subjected it to CIU using a voltage ramp in the trap collision cell prior to ion mobility separation. Interestingly a significantly higher voltage (∼25 V) was required to convert zinc-bound TFEα_TEV_ into the more unfolded state, indicating that zinc binding indeed exerted a stabilizing effect on TFEα. To gain further insight into the possible structural differences between WT TFEα_TEV_ and the C138S variant we recorded two-dimensional (2D) ^15^N–^1^H Heteronuclear Single Quantum Coherence (HSQC) spectra. The HSQC spectra of TFEα_TEV_ and the C138S variant showed good dispersion and superimposed well on each other with a single crosspeak shift between the two spectra that likely corresponds to C138 (Figure 4). These results demonstrate that (i) TFEα_TEV_ adopts a stable structure even in the absence of its interaction partner TFEβ, and (ii) that the C138S mutation does not cause larger structural perturbation of TFEα_TEV._

The addition of zinc in equistoichiometric concentrations resulted in the disappearance of a number of residues in TFEα_TEV_ (Supplementary Figure S2A), which suggests that the structure of the protein is undergoing subtle changes upon zinc binding in agreement with CIU experiments. Importantly, the set of crosspeaks disappearing in WT TFEα_TEV_ contains the single crosspeak that is perturbed in the C138S variant. Several other crosspeaks disappearing upon zinc binding in the spectra of both WT TFEα_TEV_ and the C138S variant reflecting possibly additional residues involved in zinc binding (Supplementary Figure S2A and B). It should be noted that both CIU and NMR experiments are limited to salt concentrations that are far below the physiological conditions. However, the specific changes in the HSQC spectrum upon zinc binding and the 1:1 stoichiometry observed in the native mass spectra suggest that the zinc binding is specific.

### Deletion of *tfeB* downregulates non-native genes differentially

**Figure 5.**
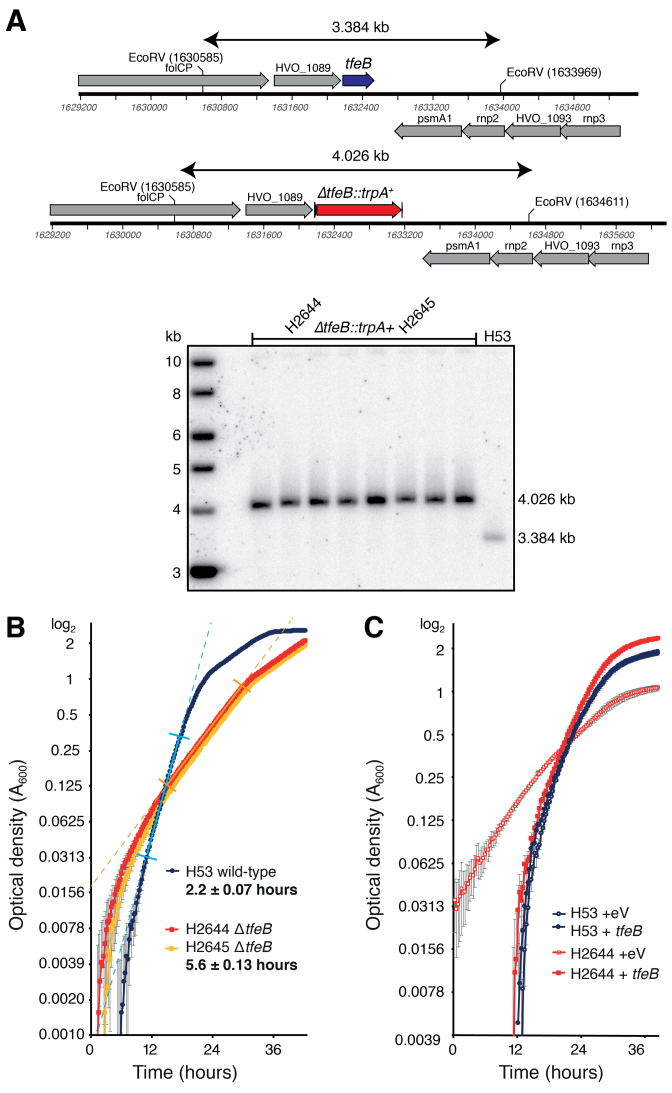
The *tfeB* gene encoding TFEβ is non-essential in *H. volcanii*. (**A**) Southern blot of *Eco*RV digest confirming deletion of *tfeB* in H2644 and H2645. (**B**) Deletion of *tfeB* confers a slow-growth phenotype in H2644 and H2645, versus the parent strain H53. Data from the mean of four repeats (and standard error) is plotted on a log_2_ scale, the generation time in exponential phase is shown in bold (exponential growth phase is indicated by straight-line fit between perpendicular marks, *R*^2^ = 0.99 for all strains). (**C**) The growth defect conferred by deletion of *tfeB* in H2644 is complemented by *in trans* expression of *tfeB* (eV, empty vector).

In order to investigate the function of TFE *in vivo*, we attempted to delete the *tfeA* and *tfeB* genes in Hvo. One key feature of *H. volcanii* as archaeal model system is its genetic tractability, which includes a ‘pop-in/pop-out’ strategy for gene deletion using tryptophan and uracil auxotrophy (23). We were unable to delete *tfeA*, despite the use of a selectable *trpA+* marker that significantly facilitates gene deletion (23), suggesting that *tfeA* is essential. However, the Hvo Δ*tfeB* strain H2644 proved to be viable but showed a severe growth phenotype, with a doubling time of 5.6 hrs compared to 2.2 h for the parental strain H53 (Figure 5A and B). Identical results were obtained with an independently-derived Δ*tfeB* strain H2645, and the growth defect in Hvo Δ*tfeB* H2644 was complemented by expression of the *tfeB* gene *in trans* (Figure 5C).

**Figure 6.**
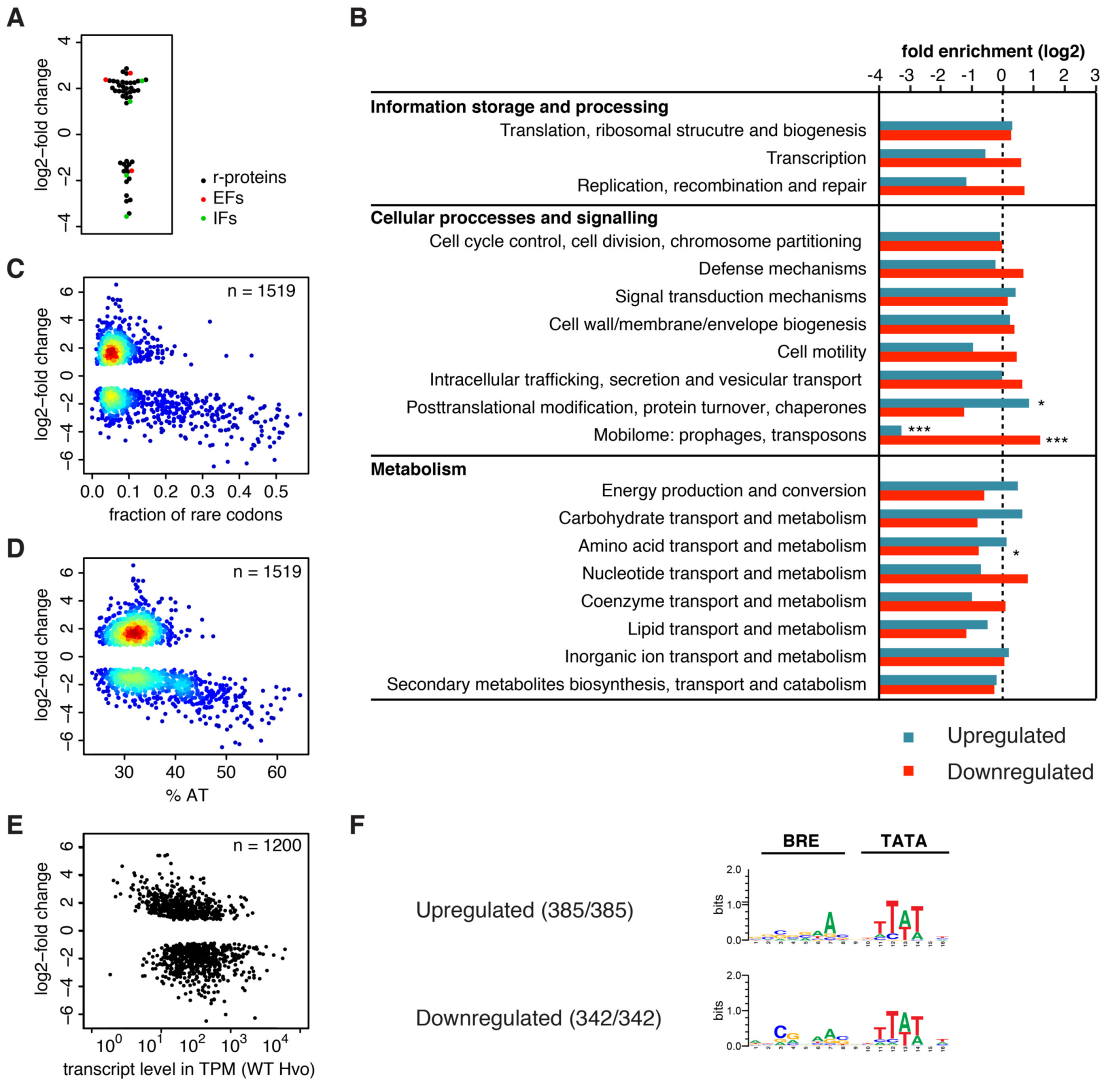
Deletion of *tfeB* leads to widespread misregulation of transcription. (**A**) log_2_-fold change in relative transcript abundance (*P*_adj_ < 0.01) of genes coding for the basal translation machinery, ribosomal proteins (black), translation initiation factors (green) and elongation factors (red). (**B**) Analysis arCOG category distribution in significantly misregulated genes (*P*_adj_ < 0.01). Significant enrichment or underrepresentation is indicated (two-sided Fisher's exact test with Bonferroni correction for multiple testing) with * and *** denoting *P*_adj_ < 0.05 and 0.001, respectively. (**C** and **D**) Heatscatter plot of rare codon frequency (defined as codons with less than 10% synonymous codon usage genome-wide) (C) and AT content of genes (D) against the log_2_-fold change (*P*_adj_ < 0.01) in relative transcript abundance. (**E**) Plot showing the estimated transcript levels in parental strain H26 (transcripts per million, TPM, geometric mean for the two replicates) calculated for the transcription units of Hvo using RSEM (31) against the log_2_-fold change in relative transcript abundance for the first cistron within the respective transcription unit (*P*_adj_ < 0.01). (**F**) BRE/TATA motif consensus in misregulated genes. In order to account for variable spacing between the TSS and BRE/TATA motifs we conducted a motif search using the MEME software version 4.11.4 (0 or 1 occurrence per sequence, 4 to 16 bp width, searching given strand only) (33). A background model based on the composition of the combined *H. volcanii* DS2 genome was employed. BRE/TATA motifs were discovered in all sequences.

The Hvo Δ*tfeB* strains provided a unique opportunity to study a putative role of TFEβ in the regulation of gene expression. In order to characterize the impact of the lack of TFEβ on the haloarchaeal transcriptome, we isolated total RNA from the Hvo Δ*tfeB* H2644 and the wild-type H26 strain. H26 is *trpA+* (23) and therefore its transcriptome will be more comparable to that of Δ*tfeB::trpA+* strain H2644 (Supplemental Table S3). RNA isolation was carried out during early exponential growth (OD_650_ = 0.2) and subjected it to next generation RNA sequencing (RNA-seq). Consistent with the predicted role of TFEβ as a basal transcription factor, the deletion of *tfeB* led to changes in relative mRNA abundance for 38% of all genes (1519 out of 3993 genes, *P*_adj_ < 0.01) (Supplemental Table S5). Approximately equal numbers of genes were up- or downregulated (781 and 738, respectively) with overall mild changes in relative transcript abundance (median values for log_2_-fold change in abundance: 1.76 and –1.86 for up- and downregulated genes, respectively). Generally, slow growth is associated with reduced ribosome biosynthesis in pro- and eukaryotes (46). This holds true independent of whether slow growth results from genetic defects or poor carbon sources. We therefore analyzed the differential expression of the components of the basal translation machinery: ribosomal proteins, initiation and elongation factors. 42 out of 57 ribosomal proteins, three out of four elongation factors, and 4 out of 13 initiation factors showed differential expression (*P*_adj_ < 0.01). The majority of these genes show increased relative abundance (32 out of 49) (Figure 6A). Therefore, the changes in the transcriptome composition appear not to reflect a general slow growth phenotype. Instead these changes most likely reflect direct misregulation of gene expression as a result of *tfeB* deletion. In order to investigate the transcriptional response more systematically, we tested for the enrichment of specific functional classes of arCOGs (40) in the sets of differentially regulated genes. Genes involved in posttranslational modification, protein turnover and folding were enriched amongst the upregulated genes while genes involved in amino acid transport and metabolism were underrepresented amongst downregulated genes (Fisher's exact test, *P*_adj_ < 0.05) (Figure 6B and Supplemental Table S6). Interestingly, genes assigned to prophages and transposons were underrepresented among upregulated, but enriched among downregulated genes (Fisher's exact test, *P*_adj_ < 0.001).

Hvo H26 harbours three megaplasmids (pHV1, pHV3 and pHV4), with pHV4 stably integrated into the chromosome (19,47). Interestingly, the knockout of TFEβ affects the genes residing on the megaplasmids (in particular pHV1) more severely compared to genes encoded by the main chromosome (Table 1). Genes encoded by pHV1differ significantly in their synonymous codon usage from the general codon usage in Hvo, suggesting that they are of non-native origin and have been acquired by lateral gene transfer (19). The notion that non-native genes are downregulated more severely than native ones is further supported by the predicted prophage residing in the main chromosome of Hvo. This region contains 43 open reading frames (ORFs HVO_2252 to HVO_2293), 32 of which have a decreased relative abundance in the Δ*tfeB* strain, while none of the remaining genes showed increased relative abundance. In order to test the effect of *tfeB* deletion on non-native genes more systematically, we plotted the rare codon frequency of genes (defined as codons with less than 10% synonymous codon usage genome-wide) against the log_2_-fold change in relative transcript abundance (Figure 6C). Many genes with high frequency of rare codons appear to be downregulated and genes with a higher AT content than the genome average showed a similar correlation (Figure 6D). The systematic comparison of genes in the first quartile to those in the fourth quartile of rare codon frequency confirmed that genes with high frequency of rare codons show a greater tendency to be downregulated. The same result was obtained when genes were partitioned based on AT content (Fisher's exact test *P* < 0.001 in both cases) (Supplementary Figure S3A).

**Table 1.** Changes in relative abundance in the Δ*tfeB* strain

	Total number of genes detected	Increased abundance	Decreased abundance
Main chromosome (excluding pHV4)*	2940	617	534
pHV1	75	1**	35**
pHV3	372	81	27**
pHV4*	606	82**	142

* Integrated into the main chromosome in laboratory strains, including H53 (47).

** *P* < 0.001 (Fisher's exact test compared to main chromosome).

To get further insight into how *tfeB* deletion globally affects the transcriptome, we calculated estimated transcript levels for the Hvo transcription units (Figure 6E). Downregulated genes appear to be associated with slightly higher transcript levels compared to upregulated ones (Wilcox rank sum test, *P* < 0.001). In summary, our results suggest that TFEβ preferentially stimulates transcription of laterally transferred (non-native) genes.

In addition to downregulated genes, the deletion of TFEβ also resulted in increased RNA expression levels that likely includes indirect effects such as a transcriptional response to suboptimal gene expression. For example, several genes encoding the basal transcription machinery including initiation factors and RNA polymerase subunits showed elevated RNA levels (Supplemental Table S5). Halophilic archaea utilize several TFB paralogues (20), which are divided into four evolutionary conserved clades (*Halobacterium* TfbA, TfbB/D/F, TfbC/G and TfbE) (48). Hvo possesses ten complete TFB paralogues. Three Hvo TFB paralogs belonging to the TfbB/D/F clade showed increased expression levels in the Δ*tfeB* strain, HVO_1052, HVO_1676 and HVO_B0285. Since TFE and TFB cooperate during OC formation, we surmise that Hvo compensates for inefficient transcription initiation due to the lack of TFEβ by increasing the expression of a subset of TFB paralogues. Haloarchaeal TFB paralogues show preferences for different BRE motifs (22). A motif search did retrieve BRE/TATA motifs with slightly altered BRE consensuses for up- and downregulated gene in the *tfeB* deletion strain, which might reflect the role of the upregulated TFB paralogues in the transcriptional response to *tfeB* deletion (Figure 6F and Supplementary Figure S3B).

Overall, our data suggest that *tfeB* deletion significantly alters the global transcriptome, particularly affecting non-native genes.

## DISCUSSION

The identification and characterization of haloarchaeal TFEβ variants provides us with a more complete picture of the evolution of TFE-like factors in archaea and eukaryotes. It corroborates the idea that dimeric TFE traces back to the last common ancestor of all archaea, and of eukaryotes. In *Euryarchaeota*, several lineages such as the *Thermococcales* and *Methanococcales* have lost the *tfeB* gene and this loss appears to coincide with a monomeric TFEα variant that seems to be functional on its own, as deducted from *in vitro* experiments (7–8,49). Other euryarchaeal lineages have retained the *tfeB* gene but we show that at least in Hvo it is non-essential. While basal transcription factors TBP and TFIIB facilitate RNAP function in eukaryotes, the archaeal homologues TBP and TFB have acquired a regulatory role, particularly in haloarchaea. We propose that the third archaeal initiation factor TFE similarly carries out a general function during OC formation and in addition stimulates transcription in a gene-specific fashion, in haloarchaea likely in conjunction with distinct TFB paralogues. The magnitude of TFE stimulation varies with the promoter context, and since the steady state levels of TFEβ in Sso rapidly decrease in response to oxidative stress and upon entering stationary phase the genes controlled by promoters that respond stronger to TFE will be down regulated significantly more compared to genes under the control of relatively unresponsive promoters (2). Importantly, the structural FeS cluster of Sso TFEβ that is crucial for all TFE functions is sensitive to oxidative damage, and its loss likely triggers the depletion of TFEβ. Interestingly the Halobacteriales have lost the FeS cluster while maintaining the C-terminal domain, an event that is paralleled in *Saccharomyces* RNAPIII subunit C34 (2,13). The loss or degeneration of metal centres in *Hvo* TFEα/β may provide insight into the apparent loss of ZR and FeS cluster domains that occurred in the evolution of hRPC62 (C82) and TFIIEβ, respectively. Similar to TFEβ, the RNA polymerase subunit D in halophilic archaea appears to have lost the structural FeS cluster (50), while other FeS cluster proteins such as XPD have retained them. What causes the loss of FeS clusters? It seems likely that general environmental factors could exert a selection pressure to drive the loss, including iron scarcity and -bioavailability in the high saline niche of haloarchaea, or elevated levels of oxidative stress in the environment. The functional adaptation of *Hvo* TFEβ is also reflected in a remarkably acidic isoelectric point. Sso TFEβ is a basic protein (predicted p*I* = 9.19) in line with the idea that its WH domain interacts with the non-template strand via electrostatic interactions on the RNAP surface during OC formation (2). In contrast, Hvo TFEβ is acidic (predicted p*I* = 4.33). Under the physiological conditions of high salinity found in the cytoplasm of *H. volcanii*, electrostatic interactions with DNA are disfavoured. In addition, high salinity directly stabilizes dsDNA by counteracting repellent forces within the phosphate backbone of the two DNA strands. While the extreme natural environments of haloarchaea are likely to have driven the adaptation of haloarchaeal TFEβ, different selective pressures might have led to the loss of the Fe-S cluster in its eukaryotic counterparts. This includes the emergence of different regulatory mechanisms for the RNAPII and III transcription machineries early in the evolution of eukaryotes. Another adaptation occurred in the *Hvo* TFEα subunit where the canonical ZR evolved into a degenerate form retaining only low affinity for zinc. This adaptation is specific to *Haloferax* and closely related species while the majority of haloarchaeal TFEα retained the four conserved cysteine residues forming canonical ZR domains. Nevertheless, it may be part of a general trend of reduced cysteine content in haloarchaeal proteomes (51,52).

The viability of the Δ*tfeB* deletion Hvo strain provided us with a unique opportunity to study the influence of TFEβ on gene expression *in vivo*. In line with its function as basal transcription factor, approximately a third of all genes were misregulated in the Δ*tfeB* strain, notably non-native genes were affected more severely than native genes. Lateral gene transfer has played a key role in the evolution of Haloarchaea both at the root and during the subsequent diversification (29,32), and the Hvo genome harbours several large clusters of non-native genes (19). The impact of *tfeB* deletion on the expression of non-native genes shows that TFEβ stimulates transcription of genes with promoters yet ill-adapted to the Hvo transcription machinery. The promoters of native genes coevolved with the host RNAP to form OCs readily including the region of the promoter that is initially melted during OC formation (IMR) and functionally interacts with TFE (2), while non-native promoters have not and apparently benefit to a greater extent from TFEβ’s ability to stimulate this process. However, this rationale is in apparent conflict with considerations concerning the negative selection pressure due to the potential harm arising from foreign genes, which are generally considered harmful (or neutral) for the organism (53). The greater dependency of these genes on TFEβ could also be caused by different chromatinization of AT-rich genes posing a stronger barrier to transcription. While we cannot rule out that the different codon usage of foreign genes during translation feeds back into transcription initiation, there is no direct evidence for such a molecular mechanism.

Bacteria including *E. coli* are known to utilize the elongation factor NusG (Spt5 in archaea) and the termination factor Rho to suppress transcription of foreign DNA (54). A rigorous sequence analysis of the promoters that were misregulated in the Δ*tfeB* strain did not provide evidence for a specific DNA sequence bias in either up- nor downregulated promoters (Supplementary Figure S3B), congruent with the hypothesis that TFEβ does not interact with DNA in a sequence specific manner. The underlying mechanisms for the promoter-specificity of TFEβ likely involves additional factors including combinations of haloarchaeal TBP/TFB variants, as well as chromatin proteins and the topology of the promoters, all of which modulate the propensity of DNA strand separation during transcription initiation.

## DATA AVAILABILITY

The accession number for the RNA-seq data reported in this paper is NCBI GEO: GSE101134.

## Supplementary Material

Supplementary Data
