# Supplementary material for: Structural and functional adaptation of Haloferax volcanii TFEα/β
Source: Nucleic Acids Res. 2018 Jan 4;46(5):2308–20. doi: 10.1093/nar/gkx1302 (PMC5861453; doi:10.1093/nar/gkx1302)
Supplement: Supplementary Data [file gkx1302_supp.zip › nar-02019-x-2017-File008.pdf]

Supplemental material

Fabian Blombach, Darya Ausiannikava, Angelo Miguel Figueiredo, Zoja Soloviev, Tanya Prentice, Mark Zhang, Nanruoyi Zhou, Konstantinos Thalassinos, Thorsten Allers, Finn Werner (2017): Structural and functional adaptation of *Haloferax volcanii* TFEα/β

This file contains supplemental tables S1-3 and supplemental figures S1-3

Table S1: Primer sequences

**Primer name**	**Sequence**	**Restriction site**	**Use**
Heterologous expression			
Hvo TFEα fw	5'-GGGCCATGGCTTTTGAGGAGTTACTGAAC-3'	*Nco*I	pRSF-1b-TFEα, pRSF-1b-TFEα-His_6
Hvo TFEα native rv	5'- GGGCTCGAGTCAGTTCGTCCCCGTCAC-3'	*Xho*I	pRSF-1b-TFEα
Hvo TFEα His_6-tag fusion rv	5'-GGGCTCGAGGTTCGTCCCCGTCACTTC-3'	*Xho*I	pRSF-1b-TFEα-His_6
Hvo TFEβ fw	5'-GGGCATATGAGCGCGAGCGAAG-3'	*Nde*I	pET-21a(+)-TFEβ, pET-21a(+)-TFEβ-His_6
Hvo TFEβ native rv	5'-GGGCTCGAGTCAGTACTCCTCGTAGGCGAG-3'	*Xho*I	pET-21a(+)-TFEβ
Hvo TFEβ His_6-tag fusion rv	5'-GGGCTCGAGGTACTCCTCGTAGGCGAGGTT-3'	*Xho*I	pET-21a(+)-TFEβ-His_6
Hvo TFEβ Δ1-72 fw	5'-GAGCATATGCCCGCCGCGCGC-3'	*Nde*I	pET-21a(+)-TFEβ Δ1-72-His_6
Hvo TFEα TEV insertion fw	5'-CACGGCTTCCAGAGCCCCGAGACGG-3'		pRSF-1b-$TFEα_{TEV}$-His_6
Hvo TFEα TEV insertion rv	5'-CCGTCTCGGGGCTCTGGAAGCCGTG-3'		pRSF-1b-$TFEα_{TEV}$-His_6
Haloferax genetic manipulation			

TFEalphaF (*tfeA* operon amplification fw)	5’-CCAGTTCGGCCTCGTACTCGCGGG-3’	(internal *Pci*I)	pTA1769
TFEalphaR (*tfeA* operon amplification rv)	5’-TCGCATGACTGAACGAACCCGCGC-3’	(internal *Kpn*I)	pTA1769
delTfeAlphaR2 (*tfeA* upstream)	5’-CAAAAGCCATgGTGCGAAATGGCGTCAGGAACGCC-3’	*Nco*I	pTA1814, pTA1858
delTfeAlphaF2 (*tfeA* downstream)	5’-GACGAACTccATGGTCGTTCTCGCAACGAAGTGC-3’	*Nco*I	pTA1814, pTA1858
TFEbetaF (*tfeB* region amplification fw)	5’-CCCCGAGATACTGTCGTCCGTCTTCG-3’	(internal *Not*I)	pTA1706
TFEbetaR (*tfeB* region amplification rv)	5’-GGAATTTCCGAGGAGAGAACCGTCG-3’	(internal *Sau*3AI)	pTA1706
delTFEbetaF (*tfeB* upstream)	5’- AGAAAGaTCTTGCTCGGCTTCGCTCGCGC-3’	*Bgl*II	pTA1708, pTA1709
delTFEbetaR (*tfeB* downstream)	5’-GAGGAGatCTGAGCCGCCCGGGGTCGC-3’	*Bgl*II	pTA1708, pTA1709

Table S2: Plasmids

**Plasmid**	**Relevant properties**	**Reference**
Heterologous expression		
pRSF-1b-TFEα,	Heterologous expression of native TFEα	
pRSF-1b-TFEα-His_6	Heterologous expression of TFEα with C-terminal His_6-fusion	
pET-21a(+)-TFEβ,	Heterologous expression of native TFEβ	
pET-21a(+)-TFEβ-His_6	Heterologous expression of TFEβ with C-terminal His_6-fusion	
pET-21a(+)-TFEβ Δ1-72-His_6	Heterologous expression of TFEβ Δ1-72 (ΔWH) variant as C-terminal His_6-fusion	
pRSF-1b-$TFEα_{TEV}$-His_6	Heterologous expression of TFEα with C-terminal TEV-cleavable His_6-fusion	
pRSF-1b-$TFEα_{TEV}$-His_6C138S	Heterologous expression of TFEα C138S variant with C-terminal TEV-cleavable His_6-fusion	
Haloferax genetic manipulation		
pTA131	Integrative plasmid based on pBluescript II, with *pyrE2* marker	(1)
pTA298	pUC19 with *trpA*$^+$ marker flanked by *Bam*HI sites	(2)
pTA354	Shuttle vector based on pTA131, with *pyrE2* marker and ori-pHV1 replication origin	(3)
pTA1706	pTA131 with *Not*I-*Sau*3AI chromosomal fragment of *tfeB* region	
pTA1708	Δ*tfeB* construct, generated by PCR of flanking regions of *tfeB* in pTA1706	
pTA1709	Δ*tfeB::trpA*$^+$ construct, generated by insertion of *Bam*HI *trpA*$^+$ fragment from pTA298	
pTA1744	pTA131 with insertion of multiple cloning site for *Nde*I, *Pci*I, *Nsp*I and *Nar*I	
pTA1769	pTA1744 with *Pci*I-*Kpn*I chromosomal fragment of *tfeA* region	
pTA1814	Δ*tfeA* construct, generated by PCR of flanking regions of *tfeA* in pTA1769	
pTA1844	pTA1744 with promoter-less *trpA*$^+$ marker flanked by *Pci*I sites	

pTA1858	Δ*tfeA::trpA*$^+$ construct, generated by insertion of *Pci*I *trpA*$^+$ fragment from pTA1844	
pTA2050	pTA354 with *Kpn*I-*Not*I fragment of pTA1706 containing *tfeB*	

Table S3: *Haloferax volcanii* strains

**Strain**	**Genotype**	**Derivation or reference**	**Use**
H53	Δ*pyrE2* Δ*trpA*	(1)	Standard laboratory strain
H2644	Δ*pyrE2* Δ*trpA* Δ*tfeB::trpA*$^{+}$	H53 pTA1709	Deletion of *tfeB*
H2645	Δ*pyrE2* Δ*trpA* Δ*tfeB::trpA*$^{+}$	H53 pTA1709	Deletion of *tfeB*
H3042	Δ*pyrE2* Δ*trpA* *tfeA*$^{+}$*::[*Δ*tfeA::trpA*$^{+}$*]*	H53 pTA1858	Transformant where Δ*tfeA:trpA*$^{+}$ could not be obtained, integrated plasmid shown in [ ]
H3043	Δ*pyrE2* Δ*trpA* *tfeA*$^{+}$*::[*Δ*tfeA::trpA*$^{+}$*]*	H53 pTA1858	Transformant where Δ*tfeA:trpA*$^{+}$ could not be obtained, integrated plasmid shown in [ ]
H3971	Δ*pyrE2* Δ*trpA {pyrE2*$^{+}$*}*	H53 pTA354	Contains empty shuttle vector
H3972	Δ*pyrE2* Δ*trpA* Δ*tfeB::trpA*$^{+}$ *{pyrE2*$^{+}$*}*	H2644 pTA354	Δ*tfeB* mutant, contains empty shuttle vector
H3974	Δ*pyrE2* Δ*trpA {tfeB*$^{+}$ *pyrE2*$^{+}$*}*	H53 pTA2050	Contains shuttle vector with *tfeB*$^{+}$ for mock complementation
H3975	Δ*pyrE2* Δ*trpA* Δ*tfeB::trpA*$^{+}$*{tfeB*$^{+}$ *pyrE2*$^{+}$*}*	H2644 pTA2050	Δ*tfeB* mutant, contains shuttle vector with *tfeB*$^{+}$ for complementation

Supplemental references

1. Allers, T., Ngo, H.P., Mevarech, M. and Lloyd, R.G. (2004) Development of additional selectable markers for the halophilic archaeon Haloferax volcanii based on the leuB and trpA genes. *Appl Environ Microbiol*, **70**, 943-953.
2. Lestini, R., Duan, Z. and Allers, T. (2010) The archaeal Xpf/Mus81/FANCM homolog Hef and the Holliday junction resolvase Hjc define alternative pathways that are essential for cell viability in Haloferax volcanii. *DNA Repair (Amst)*, **9**, 994-1002.
3. Norais, C., Hawkins, M., Hartman, A.L., Eisen, J.A., Myllykallio, H. and Allers, T. (2007) Genetic and physical mapping of DNA replication origins in Haloferax volcanii. *PLoS Genet*, **3**, e77.

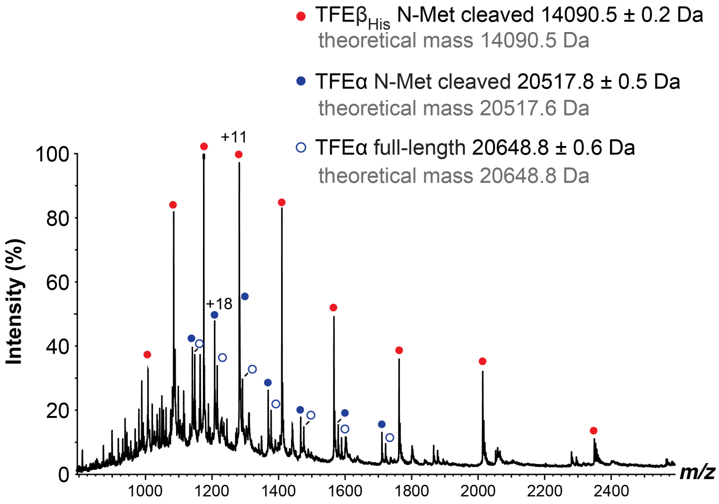

Figure S1: Mass spectrum of Hvo TFEα/β under denaturing conditions. The measured and theoretical masses for the two subunits are given. For TFEα two charge state series with a mass difference of 131 Da were obtained consistent with partial cleavage of the N-terminal methionine.

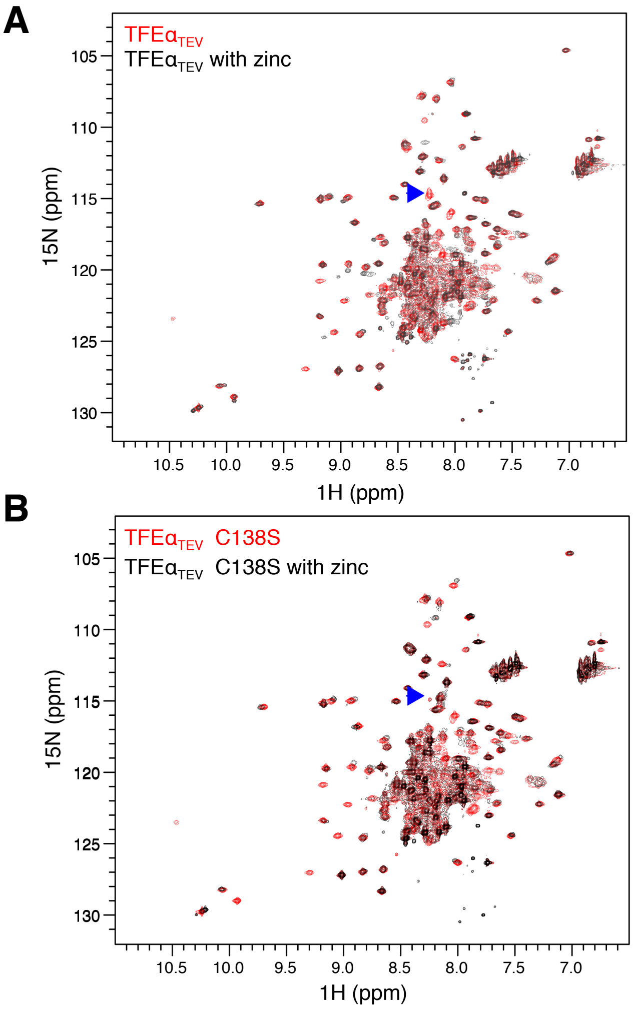

Figure S2: Zinc binding leads to conformational changes in TFEα. **(A) and (B)** Overlay of the 2D ^{15}N-^{1}H HSQC spectra of WT TFEα$_{TEV}$ (A) and the C138S variant (B) in absence (red) and presence of equistoichiometric amounts of Zn^{2+} (black). The blue arrow indicates the position of the chemical shift perturbation caused by the C138S mutation with the crosspeak disappearing in the WT TFEα$_{TEV}$ spectrum upon zinc binding. All spectra were recorded at 600 MHz in 10 mM Hepes/KOH pH 7.5, 100 mM NaCl, 1 mM DTT mixed with 7% D2O, at 25 °C.

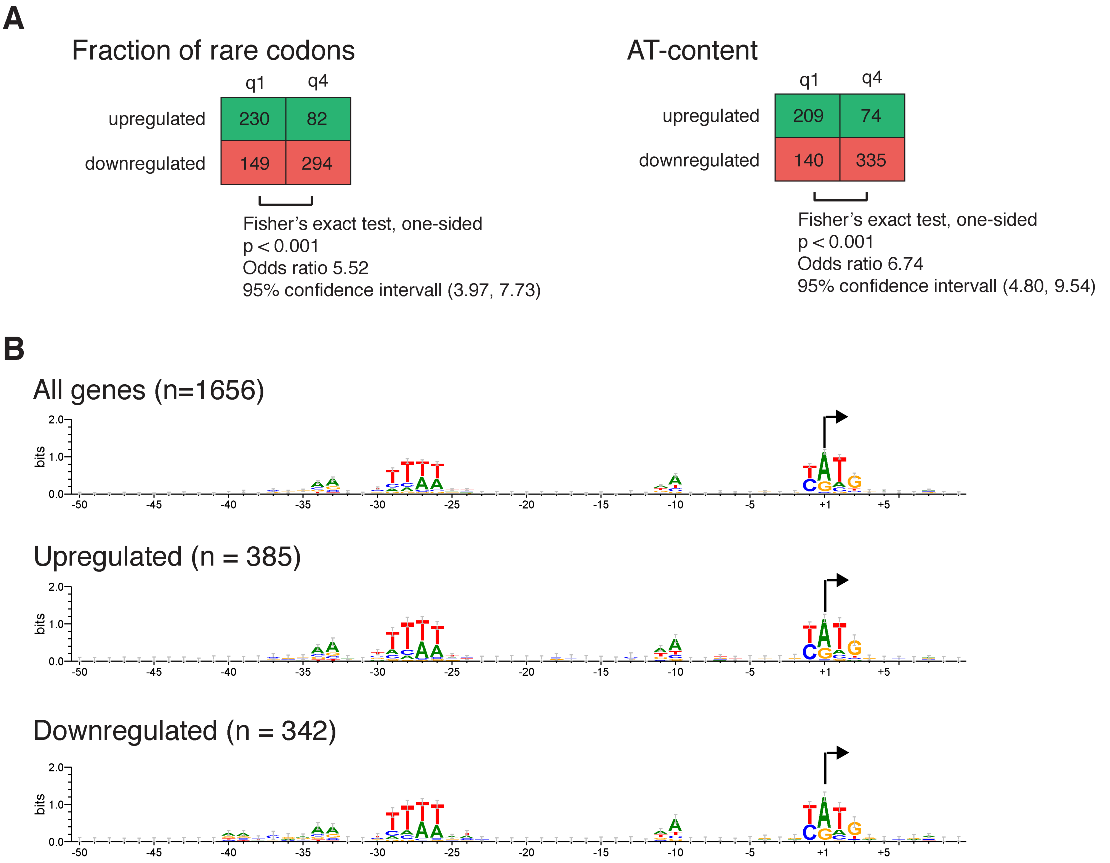

Figure S3: Analysis of misregulated genes in the Δ*tfeb* strain and their promoters. (A) Number of de-regulated genes in the first (q1) and fourth quartile (q4). Partition was based on the fraction of rare codons (upper panel) or AT content of genes (lower panel). Fisher's exact test was used to determine significance. (B) Position-specific base frequencies relative to the TSS in promoters of deregulated genes in the Δ*tfeb* strain. The logos were generated using WebLogo3 with correction for the genome composition (33).
